# Perinatal Toxoplasmosis, Northern Taiwan

**DOI:** 10.3201/eid1209.060033

**Published:** 2006-09

**Authors:** I-Jan Hu, Pau-Chung Chen, Feng-Chiao Su, Chia-Jung Hsieh, Suh-Fang Jeng, Hua-Fang Liao, Yi-Ning Su, Shio-Jean Lin, Wu-Shiun Hsieh

**Affiliations:** * National Taiwan University Hospital, Taipei, Taiwan, Republic of China;; † National Taiwan University College of Medicine, Taipei, Taiwan, Republic of China;; ‡ National Taiwan University College of Public Health, Taipei, Taiwan, Republic of China;; § National Cheng-Kung University Hospital, Tainan, Taiwan, Republic of China

**Keywords:** *Toxoplasma gondii*, seroprevalence, neonate, letter

**To the Editor:** Toxoplasmosis is caused by a protozoan parasite known as *Toxoplasma gondii*, which is found in animals worldwide and is readily transmitted to humans. The prevalence of *T. gondii*–specific immunoglobulin G (IgG) antibodies in women ranges from ≈15% in the United States (*1*) to ≈55% in Europe (*2*). Rate of transmission to a fetus in the first, second, and third trimesters is 8%, 25%, and 60%, respectively (*3*). The rate of congenital toxoplasmosis in the United States is 1–10 per 10,000 live births (*4*). Most infants infected in utero are born without obvious signs of toxoplasmosis, and learning or visual disabilities do not develop in up to 80% until their second or third decade of life (*5**,**6*).

In 1985 in Taiwan, the prevalence rates of *T. gondii*–specific IgG, as determined by ELISA, for pregnant women and their neonates were 10.2% and 11.6%, respectively. No samples from mothers or neonates were screened for IgM titers (*7*). During the past 20 years, however, the lifestyle, socioeconomic environment, and healthcare system have changed substantially in Taiwan. Overseas traveling has become more convenient, and Taiwan residents often travel to toxoplasmosis-endemic areas. The number of babies born to immigrant mothers has also recently increased in Taiwan. Our objective was to estimate the seroprevalence of perinatally transmitted *T. gondii* in northern Taiwan.

We tested sera collected from consecutive samples of women and their neonates (live births only) at 1 medical center, 1 local hospital, and 2 obstetric clinics in northern Taiwan from April 2004 through January 2005, which was 1 investigation of the Taiwan Birth Panel Study. Informed consent was obtained from either parent before enrollment in the study. Serum samples from cord blood of 483 neonates and paired samples from their mothers were analyzed for *T. gondii*–specific IgG and IgM titers by ELISA (Diagnostic Products Corporation, Los Angeles, CA, USA) (IgG sensitivity 94%, specificity 100%; IgM sensitivity 96.9%, specificity 91%) (*8*). Samples from the mothers were tested within 2 days of delivery. Additional data about health measures and conditions were collected by trained interviewers using structured questionnaires.

Among the study population, 93% were Taiwanese, 0.6% were Taiwanese aboriginals, 2.5% were mainland Chinese, and 3.9% were immigrants from southeastern Asia. Of the 483 mothers, 0.6% worked as farmers, 76% were 25–35 years of age, >50% had a university-level education, 77.7% encountered pets daily, and 9.7% owned cats. Of the 483 infants, the male:female ratio was 50.8:49.2, delivery was premature for 8.6%, and 5.8% had a low birthweight. For the mothers, the *T. gondii*–specific IgG prevalence rate was 9.1% (95% confidence interval [CI] 6.5%–11.7%), and 5 mothers (1.0%; 95% CI 0.1%–1.9%) were IgM positive. The *T. gondii*–specific IgG prevalence rate for the neonates was 9.3% (95% CI 6.7%–11.9%), and 1 neonate (0.2%; 95% CI 0%– 0.6%) was IgM positive (Table).

**Table:** Seroprevalence of toxoplasmosis in mothers and their neonates, Taiwan, 2004–2005*

Neonates	Mothers		Total
	IgG (+)	IgG (–)	
IgG (+)	43	2	45
IgG (–)	1	437	438
Total	44	439	483
	IgM (+)	IgM (–)	Total
IgM (+)	–	1	1
IgM (–)	5	477	482
Total	5	478	483

*N = 483; Ig, immunoglobulin.

We identified 2 risk factors for seropositive mothers: being from mainland People's Republic of China (odds ratio [OR] 13.42; 95% CI 1.29–19.49] and being an agricultural worker (OR 59.53; 95% CI 4.45–79.67). Incidence of positive IgG titers was higher for mothers who owned cats than for mothers who did not own cats, but not significantly higher. The neonates who had positive *T. gondii*–specific IgM had negative IgM results at the age of 3 months. No significant differences in gestational age, birthweight, and neurodevelopment were identified between seropositive and seronegative groups of infants.

This study showed that in northern Taiwan, seroprevalence of *T. gondii* among pregnant women and neonates remains low and has not substantially changed during the past 20 years. The reason for such a low incidence in Taiwan is not clear but may be attributed to differences in the lifestyle, climate, cultural differences, food habits, and to the lesser consumption of raw meat in Taiwan compared with western countries. We found that the rate for *T. gondii*–specific IgM is higher for mainland Chinese mothers than for Taiwanese mothers (p<0.05). In Taiwan, the percentage of immigrant mothers increased from 15.7% in 1998 to 32.1% in 2003, and the percentage of neonates born to these mothers increased from 5.1% in 1998 to 13.8% in 2004 (*9*). Whether the lifestyle of mothers in Taiwan with a different ethnicity will influence future *T. gondii* seroprevalence require further investigation. This study highlights the emerging importance of toxoplasmosis as a possible perinatal infection in Taiwan. A high index of suspicion for infectious diseases among immigrant mothers is needed (*10*).
